# Baltimore College of Dental Surgery Commencement

**Published:** 1899-04

**Authors:** 


### BALTIMORE COLLEGE OF DENTAL SURGERY COMMENCEMENT.

On a stage almost covered with flowers 62 bright, earnest-looking young men, composing the 59th graduating class of the Baltimore College of Dental Surgery, stood in the Lyceuu Theatre last night and received the diplomas conferred upon them by the dean ot the faculty.

The exercises were an ovation from beginning to end, and every one of the 62 who was honored with the degree of doctor of dental surgery may well be proud of the laudable encouragements showered upon him. A prayer for the class of '99 was offered by Rev. C. M. Hawkins, of Trinity M. E. Church South, and was followed by the announcement of graduates by the dean —Prof. M. W. Foster.

The conferring of degress, also by Professor Foster, was witnessed with much enthusiasm. The class marched to the front of the stage in sections of eight, and after listening to the usual reading of the authority chapter were handed the diplomas. An entirely new and very courteous custom was introduced by the graduates removing their caps and receiving the diplomas with uncovered heads. An enlivening feature of this part of the exercises was the honors and many kind remembrances given. Miss Eva P. Lemon, of Maryland, the only woman in the class. She was loudly applauded while receiving her degree, and when she reached her seat she was fairly buried under bouquets. There were at least a double dozen of fine floral offerings, and they were removed from her arms by a drayman, who took them to her home.

The annual oration before the class was delivered by Guy Carlton Lee, Ph. D., of Johns Hopkins University. It was a very able production and was listened to attentively by the class and the audience. He sought to impress them with the wisdom that life is ever a contention between discord and harmony, and that those who get the most happiness out of their labors are the ones who do their whole duty to themselves. The class valedictory was read by George W. Cheadle, of Oregon. Prof. B. Holly Smith conferred the class honors, a diamond medal, to S. Frontis, North Carolina, for highest honor, and a gold medal to C. S. McArthur, of Nova Scotia. Dr. H. H. Noble, of Washington, was to have awarded the prizes, but was prevented by sudden illness, and Dr. Adeberg, of Elizabeth, N. J., took his place.

Those who received prizes were A. J. Cohn, W. W. Cushman and J. W. Halsell in operative dentistry; F. E. Smallwood, C. R. Stewart and H. J. Smyth in mechanical dentistry; F. E. Small wood, R. W. Bickford and W. W. Barham in bridge-work, and F

S. Adams and F. E. Smallwood in orthodontia. The following were awarded honorable mention: E. B. Alkire, C. E. Gaston, J. W. Halsell, M. Douglas, W. W. Barham, S. B. Johnston, C. A. Humphreys, L. D. Coriell, F. E. Smallwood, S. P. Lee, R. W. Bickford, J. S. Lowther, G. W. Cheadle, L. R. LaFond, A. W. Leard and P. R. Stillman.

This is the graduating class: F. S. Adams, Kansas; E. B. Alkire, West Virginia; W. G. Atkinson, Massachusetts; C. W. Atter, bury, Kansas; G. W. Cheadle, Oregon; A. E. Cary, Connecticut; L. D. Coriell, Maryland; A. J. Cohn, Louisiana; A. G. Duncan-Pennsylvania; C. B. Dickson, Kentucky; W. H. Emmett, Connecticut; T. O. Flannagan, West Virginia; W. E. Gammans, Maine; A. M. German, New York; J. E. Gordon, Kentucky; C. A. Humphreys, New York; S. B. Johnston, Jr., New Jersey; E. D. Jacobus, New Jersey; F. H. James, Pennsylvania; T. D. Kelly. Jr., Kentucky; R. T. Lakin, Maryland; A. W. Leard, Canada; J. S. Lowther, Canada; G. H. Marven, Canada; D. Payne, Arkansas; E. G. Price, New York; P. R. Stillman, New York; E. B. Sefton, Maryland; C. R. Stewart, West Virginia; H. H. Street, Maryland; J. E. Taylor, South Carolina; W. W. Barham, California; W. H. Beach, Maryland; R. W. Bickford, Maryland; W. H. Bixler, Maryland; W. W. Cushman, New Hampshire; S. J. Craig, Pennsylvania; G. de Chiara, New York; M. Douglas, Canada; S. Frontis, North Carolina; C. A. Gowans, Utah; C. E. Gaston, Mississippi; H. C. Gore, Maryland; B. Holmboe, Norway; J. W. Halsell, Texas; H. H. Johnson, Novia Scotia; L. A Johnson, North Carolina; L. R. LaFond, Canada; F, H. Lambert, Canada; H. M. Leonard, Canada; S. P. J. Lee, North Carolina; C. S. McArthur, Nova Scotia; M. G. Munro, Massachusetts; T. E. McCraith, New York; A. L. Pendergrass; Arkansas; Eva E. Lemon, Maryland; C. J. Sellors, Pennsylvania; H. L. Smyth, West Virginia; F. E. Smallwood, Nova Scotia; J. P. Taggart, Ohio; W. N. Weeks, North Carolina; C. L. Woodworth, Maine.

The class officers are: President, C. J. Sellors; vice-president, E. G. Price; secretary, C. R. Stewart; valedictorian, G. W. Cheadle; class artist, W. G. Atkinson. The executive committee is composed of T. O. Flannagan, chairman; F. S. Adams, H. H. Street, A. J. Cohn and E. D. Jacobus. The orchestra was under the direction of George J. Smith, of the class of 1900.

The prizes given were: First honor, by faculty, second honor, by faculty; mechanical, by Snowden & Cowman Dental Manufacturing Company; operative, by S. S. White Dental Manufacturing Company; bridge-work, S. S. White Dental Manufacturing Company; essay prize, by Dr. J. N. Farrar, New York.

